# Transplant Trial Watch

**DOI:** 10.3389/ti.2025.14323

**Published:** 2025-01-30

**Authors:** John M. O’Callaghan, Simon R. Knight

**Affiliations:** ^1^ Centre for Evidence in Transplantation, Nuffield Department of Surgical Sciences, University of Oxford, Oxford, United Kingdom; ^2^ University Hospitals Coventry & Warwickshire, Coventry, United Kingdom; ^3^ Oxford Transplant Centre, Churchill Hospital, Oxford, United Kingdom

**Keywords:** randomised controlled trial, liver transplantation (LT), hypothermic oxygenated machine perfusion, colorectal liver metastases, chemotherapy

## Aims

The aim of this study was to perform an economic evaluation of the DHOPE-DCD trial, which investigated hypothermic oxygenated machine perfusion versus static cold preservation in liver transplant recipients receiving livers from donors after circulatory death.

## Interventions

Participants in the original trial were randomised to either receive liver preserved with hypothermic oxygenated machine perfusion following static cold preservation or with static cold preservation alone.

## Participants

156 liver transplant recipients that obtained from a donor after circulatory death that were included in the DHOPE-DCD trial.

## Outcomes

The main outcomes of interest were costs per healthcare activity, costs for machine perfusion, transplant-related healthcare costs, mean reduction in cost per patient for the 3 cost scenarios, minimal number of procedures needed per year for cost-effectiveness.

## Follow-Up

N/A.

## CET Conclusion

*by Simon Knight*

This manuscript reports an economic evaluation from the Dutch centres participating in the DHOPE-DCD randomised controlled trial. The authors looked at 3 scenarios: (1) cost for the device and consumables only, (2) costs for device and personnel, (3) costs for device, personnel and depreciation. They found that the use of D-HOPE reduced the cost of medical care in the first year post-transplant, mainly due to a reduction in ITU and intervention costs. D-HOPE achieves cost effectiveness after 30 procedures/year when personnel and depreciation costs were accounted for. This study highlights the importance of considering personnel costs, infrastructure and logistics when evaluating machine perfusion technology. In high-volume DCD transplant centres, the use of D-HOPE with a dedicated perfusion team is likely to be cost-effective, whereas in smaller volume centres it will only prove cost effective if perfusion is managed by existing staff within existing facilities. Further studies will be required to see if these findings will generalise to other healthcare settings.

## Trial Registration

ClinicalTrials.gov - NCT02584283.

## Funding Source

No funding received.

## Aims

This study aims to examine effect of liver transplantation combined with chemotherapy on overall survival among patients with permanently unresectable colorectal liver metastases.

## Interventions

Participants were randomly assigned to receive either liver transplantation plus chemotherapy or to chemotherapy alone.

## Participants

94 adult patients (18–65 years) with permanently unresectable colorectal liver metastases.

## Outcomes

The primary outcome was overall survival at 5 years. Secondary outcomes were overall survival at 3 years, progression-free survival and recurrence rate at 3 and 5 years and health-related quality of life.

## Follow-Up

5 years.

## CET Conclusion

*by Simon Knight*

This manuscript reports the outcomes from TransMet, a multicentre European open-label RCT comparing a combination of liver transplantation (LT) and chemotherapy to chemotherapy alone in patients with unresectable colorectal liver metastases and no extrahepatic disease. 94 patients were randomised, of whom 20 patients (11 in the LT and 9 in the chemotherapy group) did not receive the randomised treatment. In intent-to-treat analysis, the hazard ratio for overall 5-year survival was 0.37 (95% CI 0.21–0.65) in favour of transplantation. There were no obvious differences in the incidence of adverse events, and quality of life was similar in the two groups during follow-up. These results are impressive and suggest a significant benefit to transplantation in carefully selected patients. Methodology is good and the study is clearly reported. The findings are limited to patients with partial response or stable disease after chemotherapy, and patients with BRAF mutations were excluded. It requires prioritisation of this patient cohort in organ allocation policy to ensure expedited transplant.

## Jadad Score

3.

## Data Analysis

Strict intention-to-treat analysis.

## Allocation Concealment

Yes.

## Trial Registration

ClinicalTrials.gov - NCT02597348.

## Funding Source

Non-industry funded.

## Clinical Impact Summary

*by John O’Callaghan*

This exciting paper presents significant findings regarding the management of patients with unresectable colorectal liver metastases (CRLM). The clinical implications of this research are potentially profound.

Less than 30% of patients with CRLM are thought to be resectable. Traditionally, patients with unresectable CRLM face a poor prognosis, typically receiving chemotherapy without curative potential. This study challenges the *status quo* by exploring the role of liver transplantation not merely as a salvage procedure, but as a potential curative approach. With the increasing efficacy of chemotherapy, expertise of transplantation teams, and improvements in immunosuppression a paradigm shift in patient management is possible.

The study was a multicentre, open-label, prospective, randomised controlled trial done in 20 tertiary centres in Europe, including 94 patients randomised 1:1 between control and study arms, and stratified by centre. The liver transplantation plus chemotherapy group underwent liver transplantation within 2 months of the last chemotherapy cycle. Transplanted patients received a tailored immunosuppression regimen with postoperative chemotherapy. The control arm continued on chemotherapy. In cases of progression while on the transplant waiting list, chemotherapy was restarted, and the patient was temporarily suspended from transplantation until disease control was achieved. The primary endpoint was 5-year survival (presented in intention-to-treat and per-protocol analysis).

Intention to treat analysis showed a clinically significant difference in overall survival at 5 years: 57% for liver transplantation plus chemotherapy versus 13% for chemotherapy alone (HR 0.37 [95% CI 0.21–0.65]; p = 0.0003). The impact of liver transplantation was even greater in per protocol analysis. A similarly high proportion of patients had an adverse event in both groups (80% versus 83%).

The randomised nature of this trial, and the intention-to-treat analysis circumvents the confounding element of prior publications in this field, where patients with better prognosis may have been selected for liver transplantation over chemotherapy alone.

In summary, these findings could significantly impact clinical practice by redefining treatment pathways for patients with unresectable CRLM. This trial highlights the importance of innovative treatment strategies and the need for multidisciplinary approaches in complex cases of liver metastases.

